# The distribution of microbiomes and resistomes across farm environments in conventional and organic dairy herds in Pennsylvania

**DOI:** 10.1186/s40793-020-00368-5

**Published:** 2020-12-09

**Authors:** Dipti W. Pitta, Nagaraju Indugu, John D. Toth, Joseph S. Bender, Linda D. Baker, Meagan L. Hennessy, Bonnie Vecchiarelli, Helen Aceto, Zhengxia Dou

**Affiliations:** Department of Clinical Studies, School of Veterinary Medicine, University of Pennsylvania, Kennett Square, PA USA

**Keywords:** Antimicrobial resistance, Resistome, Metagenomics, Dairy herd

## Abstract

**Background:**

Antimicrobial resistance is a serious concern. Although the widespread use of antimicrobials in livestock has exacerbated the emergence and dissemination of antimicrobial resistance genes (ARG) in farm environments, little is known about whether antimicrobial use affects distribution of ARG in livestock systems. This study compared the distribution of microbiomes and resistomes (collections of ARG) across different farm sectors in dairy herds that differed in their use of antimicrobials. Feces from heifers, non-lactating, and lactating cows, manure storage, and soil from three conventional (antimicrobials used to treat cows) and three organic (no antimicrobials used for at least four years) farms in Pennsylvania were sampled. Samples were extracted for genomic DNA, processed, sequenced on the Illumina NextSeq platform, and analyzed for microbial community and resistome profiles using established procedures.

**Results:**

Microbial communities and resistome profiles clustered by sample type across all farms. Overall, abundance and diversity of ARG in feces was significantly higher in conventional herds compared to organic herds. The ARG conferring resistance to betalactams, macrolide-lincosamide-streptogramin (MLS), and tetracyclines were significantly higher in fecal samples of dairy cows from conventional herds compared to organic herds. Regardless of farm type, all manure storage samples had greater diversity (albeit low abundance) of ARG conferring resistance to aminoglycosides, tetracyclines, MLS, multidrug resistance, and phenicol. All soil samples had lower abundance of ARG compared to feces, manure, and lagoon samples and were comprised of ARG conferring resistance to aminoglycosides, glycopeptides, and multi-drug resistance. The distribution of ARG is likely driven by the composition of microbiota in the respective sample types.

**Conclusions:**

Antimicrobial use on farms significantly influenced specific groups of ARG in feces but not in manure storage or soil samples.

**Supplementary Information:**

The online version contains supplementary material available at 10.1186/s40793-020-00368-5.

## Background

Antimicrobial resistance (AMR) is one of the serious global public health threats of this century. It threatens the treatment and control of infections caused by microbial pathogens that are no longer susceptible to the antimicrobials commonly administered to treat them [1]. The widespread use of antimicrobials in human and animal settings has resulted in the emergence and rapid acquisition of antimicrobial resistance by pathogens, complicating disease treatments in humans and animals [2]. In 2018, the total amount of antimicrobials used in the U.S. livestock industry was approximately 11.6 million kg [3]. Of the antimicrobials approved by the Food and Drug Administration to treat livestock, 8 classes are considered to be medically relevant, meaning that they are used to treat human disease: aminoglycosides, cephalosporins, fluoroquinolones, lincosamides, macrolides, penicillins, sulfas, and tetracyclines [3]. Additionally, the World Health Organization (WHO) has developed criteria for the classification of antibiotics, based on their importance in the treatment of human disease, as “critically important,” “highly important,” and “important” [4]. The “critically important” category includes medications such as ceftiofur, a third-generation cephalosporin used in cattle to treat conditions such as bovine respiratory disease; this drug is considered critically important in human medicine because it is one of the few reliable treatments for infection with *Escherichia coli* and *Salmonella* spp. It is apparent, therefore, that the use of antimicrobials in livestock has the potential to impact human health.

As a result of the high frequency and large amounts of antimicrobials prescribed, livestock and their environment have become one of the large reservoirs of antimicrobial-resistant bacteria (ARB) and antimicrobial resistance genes (ARG) [5–7]. Notably, commensals and environmental microbes that are otherwise susceptible to antimicrobials may become resistant by acquiring ARG from resistant bacteria and thus may contribute to AMR dissemination across the food chain [8]. While the widespread use of antimicrobials has exacerbated dissemination of AMR in animals, humans, and the environment, there are only a few reports [9–11] that describe the prevalence, distribution, and dissemination pathways of ARG within agricultural sites in response to antimicrobial use on farms. How antimicrobial administration affects gut microbial populations in livestock, what resistance mechanisms are exhibited by microbiota, and whether these mechanisms disseminate to agricultural sites when voided in feces are all interesting topics for which sufficient information is lacking. Animals receiving antimicrobials induce the selection pressure such that specific bacteria that have gained resistance via mutation or horizontal gene transfer can survive [12]. Consequently, their excreta (feces and urine) can be enriched with ARG and ARB, along with antibiotic residues [13]. When manure (a mixture of feces, urine, bedding, and other materials) is subsequently applied to agricultural land, the soil then becomes a sink for the resistant pollutants [14]. The soil can also function as a source for dispersing the pollutants beyond the animal agroecosystem into and through the complex web of crops, water, wildlife, etc. [15]. Although knowledge is lacking about how and with what frequency the transfer of ARG to humans occurs, there is growing evidence that ARB and ARG from agricultural systems can affect humans through numerous routes [16–18].

Previous studies have investigated the fate of a limited number of ARG in farm sectors [19, 20]. In a previous study [21], we have demonstrated the distribution of ARG in feces, manure, and soil across dairy agroecosystems and identified a diverse pool of ARG in manure. Metagenomic approaches offer the possibility of exploring the complete spectrum of ARG within a microbial ecosystem [13] and have been increasingly applied to survey livestock [22] and the broader livestock environment [13, 23] to expand our understanding of the distribution of ARB and ARG in animal production systems. The purpose of this study was to determine whether antimicrobial use on dairy farms influences microbial community structure and composition and distribution of antimicrobial resistant genes in feces, manure storage, and soil in dairy agroecosystems. Our hypothesis was that antimicrobial use on farms would differentially affect the prevalence and distribution of ARG in different farm sectors. To test this, we compared certified-organic (antimicrobials were not used to treat animals for at least 4 years) and conventional herds (antimicrobials have been used to treat animals) matched by farm size for the distribution of ARG using metagenomic approaches.

## Methods

### Selection of dairy herds and sample collection

A list of certified organic dairy operations was obtained from the USDA Agricultural Marketing Service (https://www.ams.usda.gov/services/organic-certification/certifying-agents) from which 3 organic dairy herds were selected and matched with 3 conventional dairy herds based on size and geographic region. Five herds were located in southeastern Pennsylvania and 1 herd was located in northeastern Maryland. Herds were classified and matched by size based on the total number of mature cows on farm. Herd size classification was: i) small herds of 40–100 cows, ii) medium herds of 101–300 cows, and iii) large herds of 301–500 cows. The actual herd sizes were as follows: small organic – 40 cows, small conventional – 72 cows, medium organic – 190 cows, medium conventional – 210 cows, large organic – 500 cows, and large conventional – 490 cows.

### Sampling of cow feces

The design of the experiment and animal sampling protocol were approved by the University of Pennsylvania Institutional Animal Care and Use Committee (IUCAC protocol number 806194). All animal sampling was conducted on private farms with the permission of the farm owners. Fecal samples were collected via rectum from 3 lactating cows, 3 non-lactating cows, and 3 heifers between the ages of 6–12 months for each of the 6 farms. Fecal samples were individually collected and placed in 50 mL conical tubes. About 250 g of sample was collected for each sample type. Within 2 h of sampling, the samples were transported on ice to the lab where they were archived at − 80 °C. All samples were extracted for DNA in one batch.

### Sampling of manure storage

Manure storage samples were obtained from farm-specific manure collection systems. All farms utilized a permanent manure storage structure (lagoon or concrete pit) to store all or a portion of manure. A container was lowered into the manure storage structure to collect manure samples that were composited for analysis. On farms that stacked manure deposits as a pile, a composite of the pile was sampled to represent a manure sample. The manure sample was transferred into 50 mL conical tubes, placed on ice and transported to the laboratory along with fecal samples.

### Sampling of soil

Soils from the 3 organic and 3 conventional dairy farms were sampled with a stainless-steel, 1.6 cm diameter probe. Twenty to 30 soil cores were taken in a grid pattern in a representative area of the pastures (2 farms) and crop fields (4 farms) and these samples were composited for analysis. Samples were collected between 8 March and 26 June 2017. The most recent manure applications to these fields occurred in fall 2016, prior to planting the winter cover crop. In addition, soil samples, one from a pristine forest and the other from an agricultural experimental field that has had no known manure application, served as comparisons. Bulk soil samples were crumbled and air-dried, then sieved to pass through a 2 mm mesh screen and stored at − 20 °C until use in the laboratory.

### Sample processing and sequencing

The genomic DNA was extracted from all samples using PowerSoil DNA Isolation Kit (MOBIO Laboratories, Inc., Carlsbad, CA), as described by Pitta et al. [21]. Library preparation for shotgun sequencing was performed using Nextera XT DNA Library Preparation Kit (FC-131-1024, Illumina, San Diego, CA, USA) following the manufacturer’s protocol. Briefly, 1 ng of extracted DNA from each sample was subjected to tagmentation followed by addition of indexes and adapters using a limited-cycle PCR program. The generated libraries were cleaned using AMPure XP beads (Beckman Coulter; Brea, CA), normalized, and pooled. Shotgun sequencing was performed on the Illumina NextSeq500 platform (tight insert size of 250 bp for high-throughput sequencing from both ends by 2 × 150 bp).

### Bioinformatics analysis

Raw sequences were subjected to quality trimming using Trimmomatic (0.36) [24] according to the following parameters: starting from either end of the sequence, bases were trimmed off if their Phred quality score was < 3 or if they appeared as N; bases were trimmed off if their average Phred quality score was < 15 when the sequence was analyzed on a 4-base sliding window; and sequences were removed if they were shorter than 36 bases in length. Reads aligning to the host genome (ARS-UCD1.2/bosTau9) were identified and removed using Bowtie2 (v2.2.7) [25] with parameters set by the flag “--very sensitive local --un-conc”.

To identify ARG sequences, quality-controlled reads were aligned to MEGARes 2.0 (a hand-curated antimicrobial resistance genes database containing 7868 ARG) [26], using BWA v0.7.13 [27]. The aligned (BAM) files were then sorted and converted to a SAM file using Samtools v1.7 [28]. The MEGARes database contains specific annotation of genes (sequence headers contain ‘RequiresSNPConfirmation’) that require the presence of single nucleotide polymorphisms (SNP) at specific loci in order to confer resistance [29]. Therefore, alignments labeled with “RequiresSNPconfirmation” in the SAM-formatted file were extracted into a single fasta file which was further subjected to a secondary validation analysis by using Resistance Gene Identifier (RGI) (v 5.1.1) with “Perfect” and “Strict” algorithms [30]. Sequencing reads that did not survive the RGI-based confirmation were excluded from the SAM file, and the remaining alignments were then analyzed through ResistomeAnalyzer (−t 80; at least 80% of nucleotides in the reference sequence that were aligned to by at least one sequence read) to parse the number of resistance genes within each sample (https://github.com/cdeanj/resistomeanalyzer). We also performed at 100% gene comparison (Additional Table 5) but presented all our comparisons at 80%.

Taxonomic labels were assigned to quality-controlled reads by mapping sequences to a low-complexity masked database of bacterial, archaeal, viral, fungal, and protozoal sequences from NCBI complete genomes (downloaded 22 October 2020). The relative abundance of the bacterial genus was estimated using Bracken [31]. In order to identify the bacterial hosts (ARB) of identified ARG, for each sample, the kraken2 output file, which contains the sequence read ID and the assigned taxonomy label, was merged with each ARG output file using the sequence read ID as the key in R. The combined output was used to assign ARG to their corresponding bacterial hosts.

The taxonomy and ARG sequence abundances were normalized to the counts per million approach accounting for various sequence depths across samples using the ‘cpm’ function available in edgeR library in R. The Bray-Curtis dissimilarity index was calculated on the normalized data and was visualized using non-metric multidimensional scaling (NMDS) using the metaMDS function available in vegan R package. A non-parametric permutational multivariate analysis of variance test (PERMANOVA), implemented in the vegan package for R [32], was used to test the effects of farm type (conventional or organic), sample type (feces, lagoon, manure, or soil), and the interactions of these variables on overall community composition. PERMANOVA tests were done on Bray-Curtis dissimilarity index. Comparisons of ARG (cpm) between farm type, sample type and their interactions were tested using generalized linear model.

## Results

### Sequencing information

In this study, a total of 33 metagenomic libraries inclusive of cow feces, manure, lagoon, and soil samples (hereafter referred to as sample type) collected from 3 conventional and 3 organic farms (hereafter referred as farm type) in southeastern Pennsylvania were analyzed for their composition of microbial communities, distribution of ARG, and bacterial hosts carrying ARG. Antibiotic use on conventional farms is reported in Additional Table 1.

A total of 1 billion paired end reads were sequenced. These raw sequences were trimmed and then quality filtered (about 5% of reads were eliminated; 4% Trimmomatic filtering and 1% host filtering) resulting in approximately 971 million reads (Additional Table 2). The average number of sequence reads per sample was 29,346,035 (min: 8,135,779-max: 112,217,060) A total of 9123 ARG were identified by comparing quality-filtered sequence reads to the MEGARes2 database.

### Community clustering patterns based on microbiome and resistome profiles

**Fig. 1:**
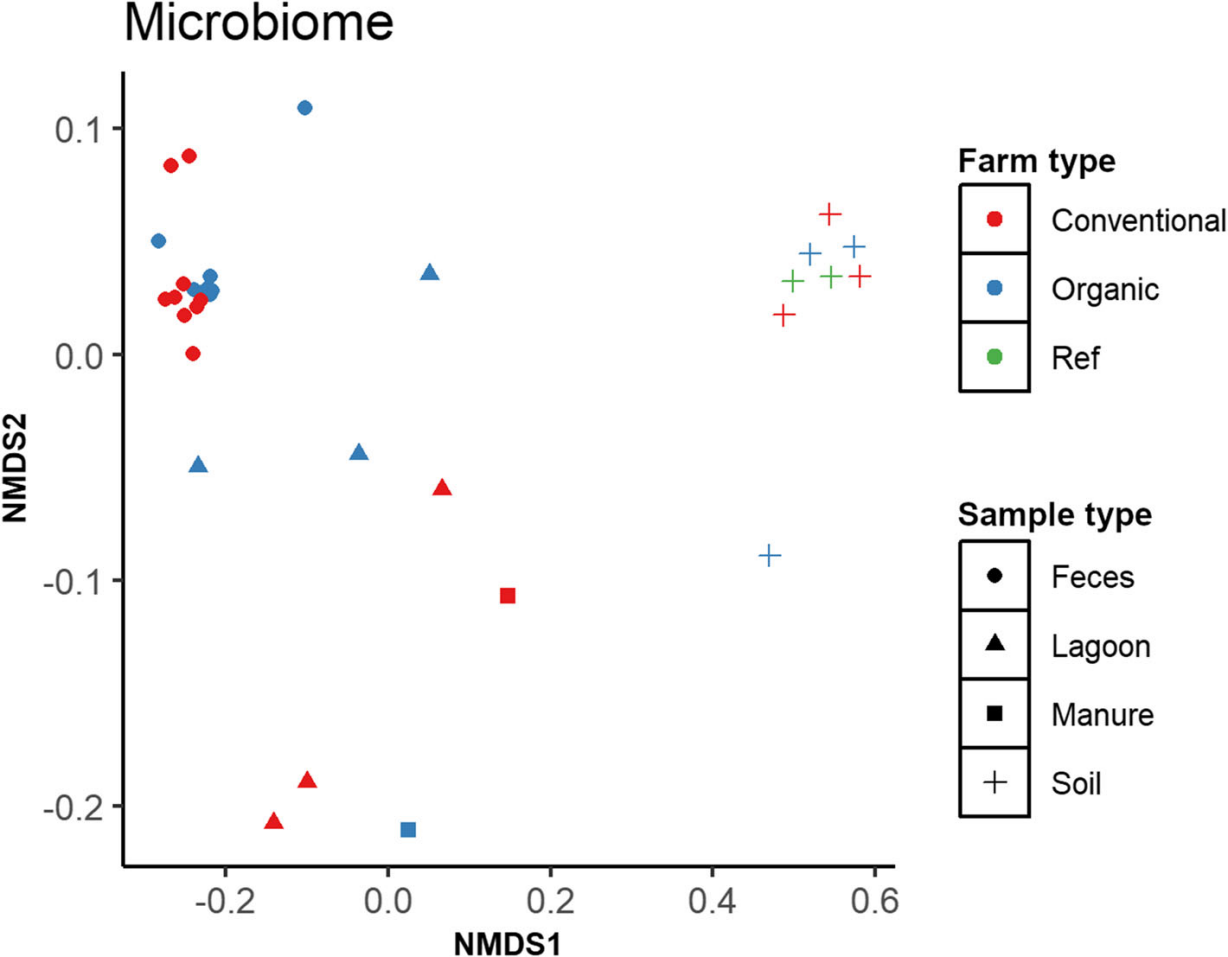
Community clustering patterns of microbiomes. Nonmetric multidimensional scaling (NMDS) ordination plot based on Euclidean distances calculated for each pair of samples and depicted by farm and sample types using the bacterial genus level information of metagenomic data. Ref: reference

Based on nonmetric multidimensional scaling (NMDS) analysis (Fig. 1), microbial communities clustered by sample type irrespective of farm type. Within each sample type, manure and lagoon microbiomes between different farms showed greater variation whereas fecal and soil microbiomes remained homogenous except for one soil sample. Interestingly, the soil microbiome in reference soil samples (i.e. soils from the pristine forest as well as the experimental field receiving no manure) was similar to those soil samples collected on dairy farms.

**Fig. 2:**
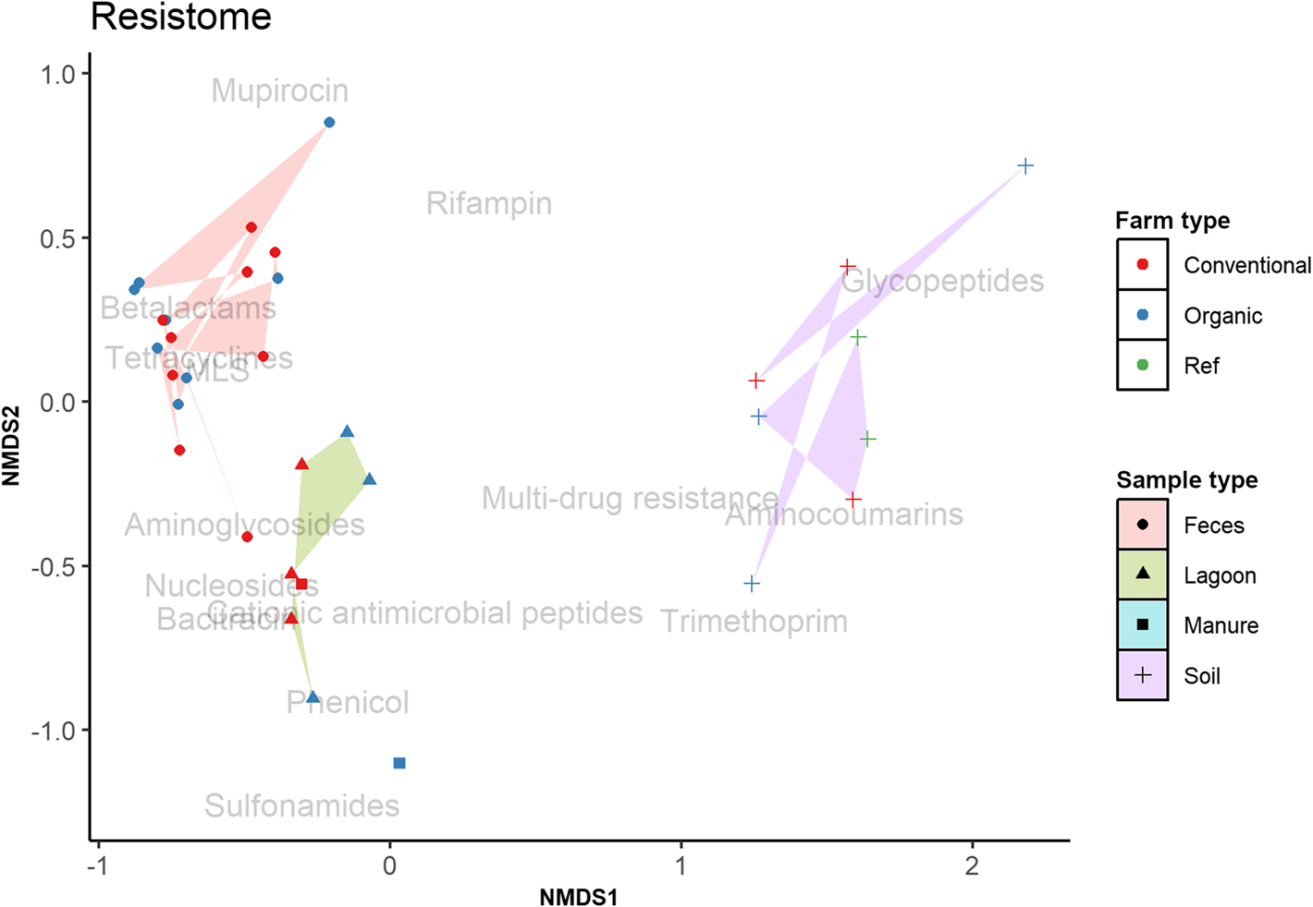
Community clustering patterns of resistomes. Nonmetric multidimensional scaling (NMDS) ordination plot of resistomes of conventional and organic farms. Each of the four polygons represents a sample type. Ref: reference

Resistomes (collections of ARG) also clustered by sample type and were consistent with the clustering patterns of microbiomes (Fig. 2). In fecal samples of both farm types, resistomes were predominated by ARG that conferred resistance to tetracyclines, MLS, and betalactams. Across all manure and lagoon samples, resistomes were diverse and contained ARG that conferred resistance to several antimicrobials. Soil resistomes, including reference soil samples, clustered together with ARG that conferred resistance to aminocoumarins, glycopeptides, and multidrug resistance.

Based on PERMANOVA (Additional Table 3), no differences in microbiomes and resistomes were observed between organic and conventional herds but significant differences were observed between sample types (*P* < 0.05).

### Microbial community composition

A total 37 different bacterial phyla were identified across all samples (Table 1). The most dominant phyla were *Proteobacteria* (35%), *Firmicutes* (22%), *Bacteroidetes* (21%), and *Actinobacteria* (16%) that collectively contributed to the greatest bacterial abundance. Fecal samples were dominated by *Firmicutes* (37%) and *Bacteroidetes* (31%) followed by *Proteobacteria* (17%) and *Actinobacteria* (10%) across both conventional and organic dairy cows. *Proteobacteria* constituted the majority (57%) of bacterial abundance in manure and lagoon samples across both farm types whereas *Proteobacteria* (55%) and *Actinobacteria* (35%) together comprised the majority of bacterial populations in soil samples of both farm types.

**Table 1:** Mean relative abundance (%) of bacterial phyla calculated based on copies per million reads across feces, manure, lagoon and soil samples from both farm types

Phylum	CF	CL	CM	CS	OF	OL	OM	OS	RS	Overall
Proteobacteria	16.78	59.72	63.16	53.06	17.38	42.58	63.86	57.17	56.77	35.40
Actinobacteria	8.18	8.50	13.30	37.31	11.93	9.40	11.33	32.51	33.65	15.88
Bacteroidetes	34.36	19.40	12.07	2.24	27.67	19.27	12.29	4.05	2.27	21.04
Planctomycetes	0.17	0.19	0.36	2.09	0.22	0.37	0.13	1.90	2.25	0.67
Firmicutes	35.06	8.37	7.05	2.13	38.06	16.77	9.69	1.74	1.74	22.04
Acidobacteria	0.14	0.09	0.15	0.95	0.15	0.17	0.06	0.78	1.01	0.32
Gemmatimonadetes	0.02	0.03	0.11	0.52	0.03	0.06	0.04	0.36	0.50	0.14
Verrucomicrobia	0.30	0.10	0.44	0.35	0.39	0.64	0.09	0.33	0.53	0.35
Deinococcus-Thermus	0.14	0.11	0.20	0.31	0.19	0.21	0.25	0.30	0.29	0.20
Cyanobacteria	0.70	0.35	0.32	0.32	0.81	0.96	0.29	0.28	0.32	0.60
Chloroflexi	0.15	0.18	0.28	0.25	0.19	0.61	0.09	0.21	0.20	0.23
Nitrospirae	0.04	0.02	0.03	0.16	0.05	0.06	0.02	0.11	0.15	0.06
Kiritimatiellaeota	0.01	0.03	0.02	0.06	0.02	0.17	0.01	0.05	0.06	0.04
Spirochaetes	1.48	1.54	0.76	0.05	1.04	5.46	0.14	0.04	0.05	1.33
Chlorobi	0.12	0.08	0.06	0.04	0.14	0.21	0.04	0.04	0.05	0.11
Armatimonadetes	0.01	0.01	0.01	0.03	0.01	0.01	0.00	0.02	0.03	0.01
Synergistetes	0.08	0.13	0.05	0.03	0.09	0.32	0.02	0.03	0.02	0.09
Unclassfied	0.01	0.01	0.07	0.01	0.02	0.04	0.01	0.01	0.01	0.02
Thermotogae	0.13	0.08	0.12	0.01	0.14	0.23	0.04	0.01	0.01	0.10
Tenericutes	0.68	0.73	0.75	0.01	0.67	1.69	1.17	0.01	0.01	0.63
Fusobacteria	0.73	0.14	0.11	0.01	0.38	0.27	0.17	0.01	0.01	0.34
Candidatus Bipolaricaulota	0.00	0.00	0.00	0.01	0.00	0.01	0.00	0.01	0.00	0.00
Aquificae	0.06	0.02	0.01	0.01	0.08	0.04	0.01	0.01	0.01	0.04
Chrysiogenetes	0.01	0.00	0.01	0.01	0.01	0.01	0.00	0.01	0.01	0.01
Chlamydiae	0.07	0.03	0.03	0.01	0.08	0.04	0.03	0.00	0.01	0.05
Thermodesulfobacteria	0.03	0.01	0.01	0.00	0.03	0.03	0.01	0.00	0.00	0.02
Balneolaeota	0.01	0.01	0.01	0.00	0.01	0.02	0.01	0.00	0.00	0.01
Deferribacteres	0.04	0.02	0.03	0.00	0.04	0.04	0.01	0.00	0.00	0.03
Calditrichaeota	0.01	0.01	0.00	0.00	0.01	0.02	0.00	0.00	0.00	0.01
Ignavibacteriae	0.02	0.01	0.01	0.00	0.02	0.03	0.01	0.00	0.00	0.02
Elusimicrobia	0.03	0.01	0.00	0.00	0.04	0.03	0.00	0.00	0.00	0.02
Fibrobacteres	0.04	0.03	0.02	0.00	0.06	0.05	0.01	0.00	0.00	0.03
Caldiserica	0.00	0.00	0.00	0.00	0.00	0.00	0.00	0.00	0.00	0.00
Coprothermobacterota	0.00	0.00	0.00	0.00	0.00	0.00	0.00	0.00	0.00	0.00
Candidatus Saccharibacteria	0.01	0.00	0.00	0.00	0.01	0.02	0.01	0.00	0.00	0.01
Candidatus Cloacimonetes	0.38	0.03	0.42	0.00	0.01	0.15	0.15	0.00	0.00	0.14
Dictyoglomi	0.01	0.00	0.00	0.00	0.01	0.01	0.00	0.00	0.00	0.01

*CF* conventional feces; *OF* organic feces; *CL* conventional lagoon; *OL* organic lagoon; *CM* conventional manure; *OM* organic manure; *CS* conventional soil; *OS* organic soil; *RS* reference soil

### Identification and characterization of ARG

**Fig. 3:**
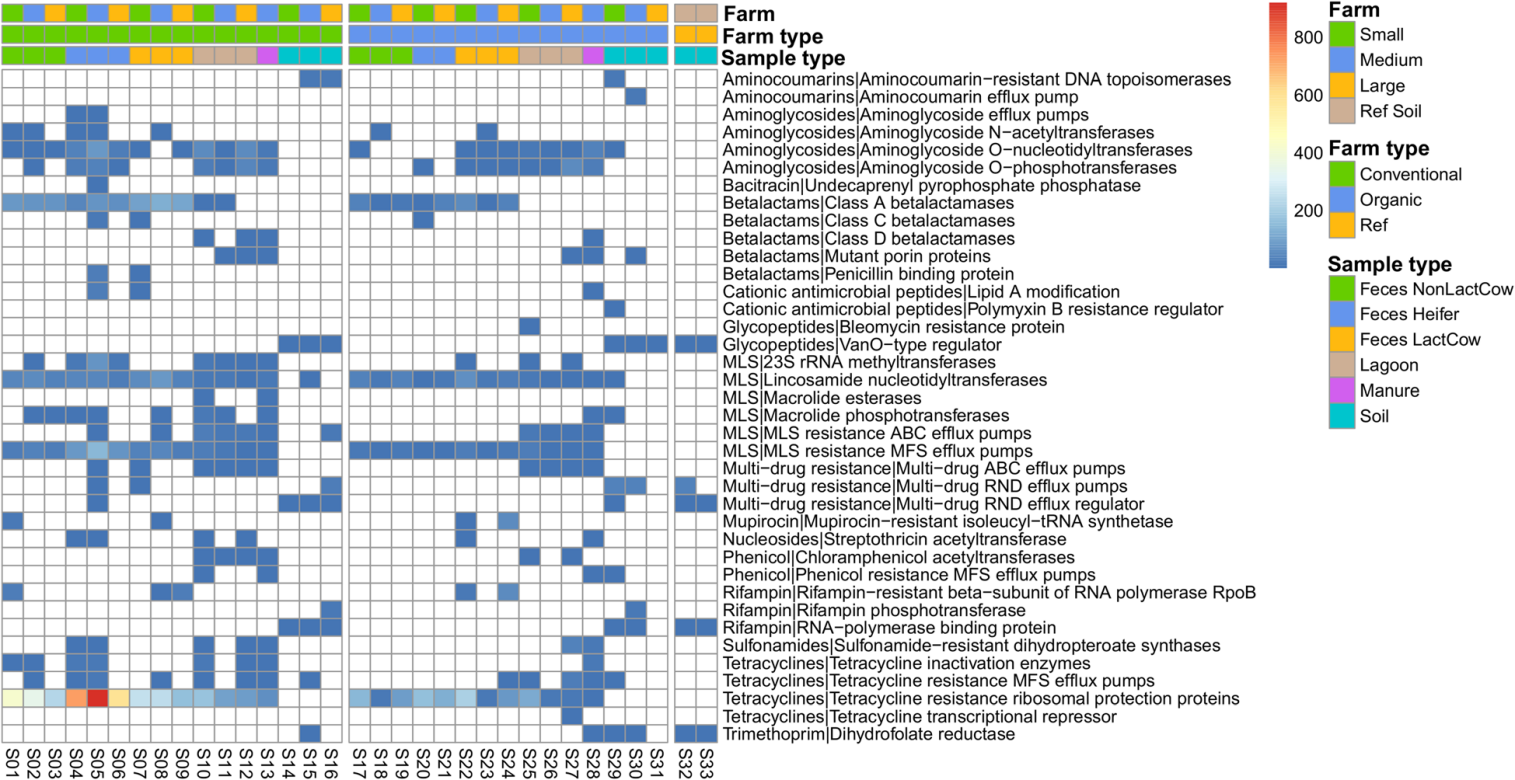
Heat map of resistance mechanisms. Heat map showing resistance mechanisms for each class of antimicrobials identified in fecal, lagoon, manure, and soil sample types of conventional and organic farms. Relative abundance of each mechanism in each sample is given by the color code in upper right-hand corner of figure. Ref: reference; LactCow: lactating cow; NonLactCow: non-lactating cow

A total of 401 unique ARG were identified from 9123 sequences across all samples (Additional Table 4). The means of ARG (CPM) conferring resistance to different classes of antimicrobials across different sample types in both farm operations are presented in Table 2. For each class of antimicrobials, the type of resistance mechanism (Fig. 3) and the corresponding gene(s) are also presented (Additional Table 4). Overall, 38 different resistance mechanisms conferring resistance to 15 classes of antimicrobials were described. Of the 401 unique ARG, 305 representing 34 different mechanisms were identified on conventional farms, whereas 307 unique ARG representing 34 different mechanisms were identified on organic farms. The total number of ARG identified in conventional herds was 7020 while on organic farms, 2048 ARG were identified. Only 3 unique ARG were identified in pristine soil environments used as controls (soil from an agricultural field that had never received manure application and forest soil, respectively).

**Table 2:** Average distribution of antimicrobial resistance genes (copies per million) at class level in all sample types across both farm types

	Conventional farms				Organic farms				Reference	Significance – P values		
	CF	CL	CM	CS	OF	OL	OM	OS	RS	FT	ST	FT:ST
Aminocoumarins	0.00	0.00	0.00	21.98	0.00	0.00	0.00	22.60	0.00	0.940	0.466	0.927
Aminoglycosides	220.54	176.20	32.76	0.00	22.51	75.26	57.79	0.21	0.00	0.356	0.564	0.716
Bacitracin	4.50	0.00	0.00	0.00	0.00	0.00	0.00	0.00	0.00	0.526	0.564	0.741
Betalactams	751.60	18.07	6.02	0.00	180.36	1.28	4.61	6.25	0.00	0.000	0.001	0.042
CAP	23.48	0.00	0.00	0.00	0.00	0.00	1.13	5.46	0.00	0.356	0.529	0.562
Glycopeptides	0.00	0.00	0.00	20.73	0.00	0.10	0.00	17.81	12.44	0.922	0.015	0.741
MLS	983.59	158.17	30.21	10.71	271.03	80.99	12.65	2.46	0.00	0.008	0.015	0.188
Multi-drug resistance	23.51	9.57	2.55	23.76	0.00	7.14	0.79	63.64	41.10	0.356	0.564	0.188
Mupirocin	23.32	0.00	0.00	0.00	54.95	0.00	0.00	0.00	0.00	0.690	0.980	0.741
Nucleosides	10.61	1.21	0.00	0.00	0.21	0.00	0.12	0.00	0.00	0.356	0.529	0.716
Phenicol	0.00	3.64	2.05	0.00	0.00	1.09	6.45	0.44	0.00	0.922	0.980	0.741
Rifampin	52.48	0.00	0.00	15.13	61.37	0.00	0.00	16.28	1.83	0.922	0.980	0.927
Sulfonamides	2.06	12.77	3.98	0.00	0.00	32.30	11.43	0.00	0.00	0.922	0.980	0.927
Tetracyclines	3942.03	365.02	67.56	0.47	838.18	165.77	18.92	4.37	0.00	0.005	0.013	0.186
Trimethoprim	0.00	0.00	0.00	0.15	0.00	0.00	1.47	0.44	0.23	0.922	0.642	0.562

*CF* conventional feces; *OF* organic feces; *CL* conventional lagoon; *OL* organic lagoon; *CM* conventional manure; *OM* organic manure; *CS* conventional soil; *OS* organic soil; *RS* reference soil; *FT* Farm type; *ST* Sample type; *CAP* Cationic antimicrobial peptides

### ARG profiles in different sample types of organic and conventional dairy systems

Overall, feces sampled from animal groups in conventional herds had a greater abundance as well as more diverse ARG compared to feces from animal groups in organic herds (Fig. 3; Additional Table 4). There was an abundance of ARG conferring resistance to aminoglycosides, betalactams, tetracyclines (ribosomal protection proteins), and macrolide-lincosamide-streptogramin (MLS; lincosamide nucleotidyltransferases) across feces from all animal groups from both organic and conventional herds. However, these ARG were significantly more abundant in conventional herds compared to organic herds. In organic herds, heifers and non-lactating cows did not carry any additional ARG in their fecal samples. The fecal samples of lactating cows in large and medium organic herds had an abundance of ARG conferring resistance to rifampin (*rpoB*) whereas feces from lactating cows in the small organic herd had a very low abundance of ARG conferring resistance to class A betalactamases and aminoglycoside-O-phosphotransferases (AG-O-PT). Notably, in the conventional herds, ARG conferring resistance to class A betalactamases (*cfx*) were detected in feces from all animal groups. The feces from the heifer group in the medium conventional herd had multiple ARG including those conferring resistance to class A betalactamases (*cblA, ctx, rob*), class C betalactamases (*bLaEC*), cationic peptides, fluoroquinolones, MLS, multidrug resistance, and tetracyclines (*tetX, tet32, tet44, tet40, tetQ,* and *tetR*). The feces from the non-lactating and lactating cows in the medium herd did not have any additional ARG except those that were common to all fecal samples in conventional herds. Feces from the heifers from the small conventional herd also had diverse ARG but in low abundance.

Regardless of the farm type, the manure and lagoon samples were predominated by ARG conferring resistance to tetracyclines, MLS, and aminoglycosides. Although the same type of ARG was detected in the manure and lagoon samples of both farm types, their abundance was higher in conventional farms compared to organic farms; however, there was a large variation between farms. The ARG conferring resistance to class D betalactamases (*blaOXA*) was present in 3 of 4 samples in the conventional herd whereas it was present only in the medium organic herd but in very low abundance.

The soil ARG were distinct from feces and manure, with soil being predominated by ARG conferring resistance to aminocoumarins, aminoglycosides, glycopeptides, and multi-drug resistance. The overall abundance of soil ARG was much lower than feces and manure but very similar between organic and conventional herds. The ARG profiles of soils collected on farms were similar to forest soil.

### Taxonomy associated with antimicrobial classes

**Fig. 4:**
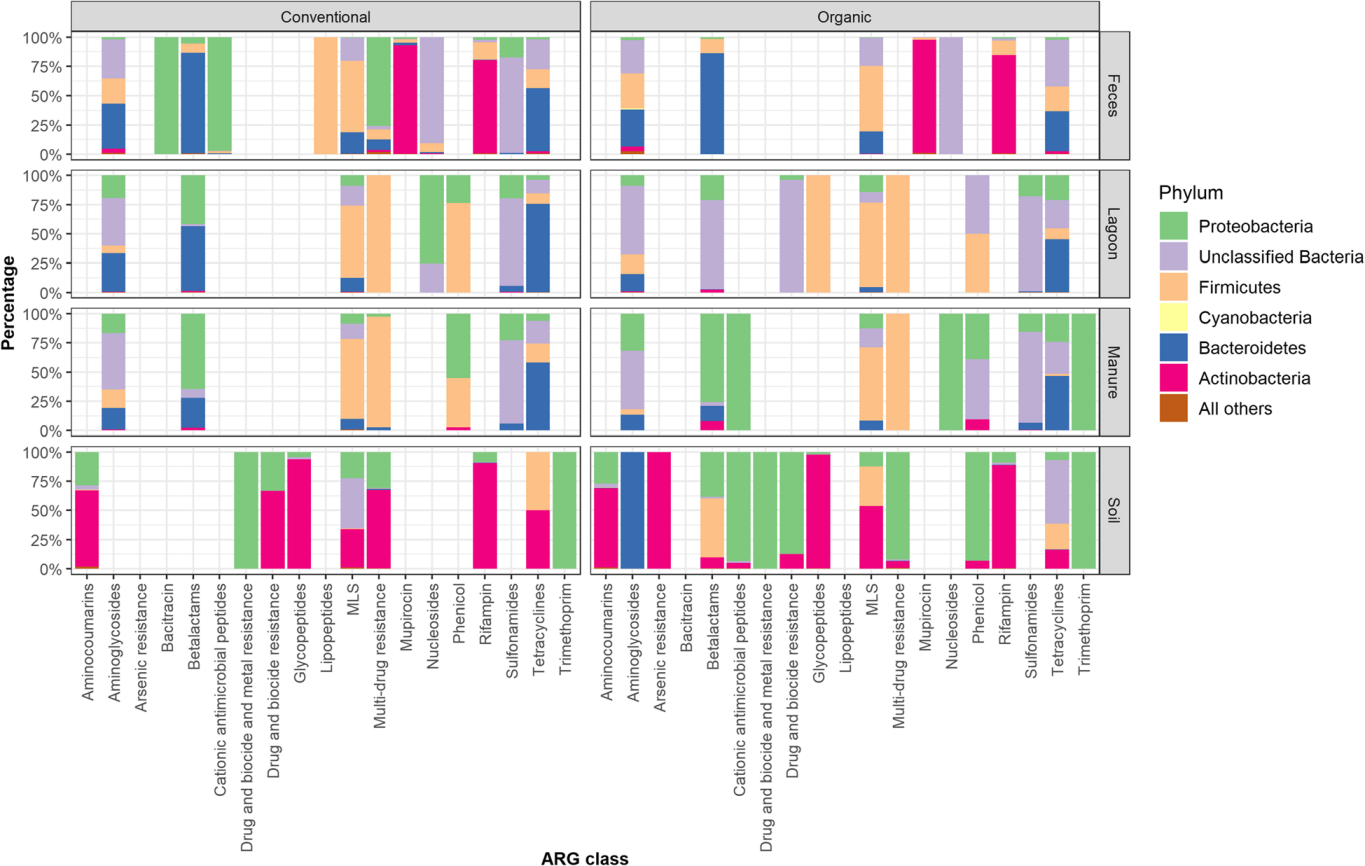
Bacterial hosts carrying ARG. Stacked bar plot depicting the percent contribution of bacterial hosts (identified to the phylum level) carrying ARG (antimicrobial resistance genes) for different classes of antimicrobials in each farm type

The resistance-encoding ARG were mainly harbored in lineages belonging to the bacterial phyla *Actinobacteria*, *Bacteroidetes*, *Firmicutes*, and *Proteobacteria* (Fig. 4); those ARG for which the bacterial host was not identified were grouped as unclassified phylum (about 23% of ARG reads were unclassified bacteria).

Among the aminoglycoside ARG, the bacterial hosts for efflux pump resistance mechanisms were exclusive to *Proteobacteria*. Among the aminoglycoside-N-acetyltransferases, the *aac3* ARG was abundant in the dairy cow feces from the medium conventional farm. The host carrying this gene was the *Enterobacteriaceae* family. The *aac(6′)* ARG was present in dairy cow feces across both farm types and was carried in almost all bacterial lineages. While most of the bacterial hosts carrying aminoglycoside-O-nucleotidyltransferase (AG-O-NT) genes were not identified, a few of the genes for AG-O-NT, particularly *ant(3′)*, were distributed in manure and lagoon samples and were mostly carried by *Proteobacteria* lineages. However, *ant6* was distributed in fecal samples and was carried by *Clostridia* and *Bacilli* from *Firmicutes*, *Actinomycetacea*e from *Actinobacteria*, and a few *Bacteroidetes* members. The *ant6* identified in soil samples from the small organic herd was carried by *Bacteroidetes* members only. Interestingly, *ant9* genes were distributed in fecal and manure samples of both herds and were predominantly carried by *Bacteroides* members. The acetyl-O-phosphotransferases (AG-O-PT) were distributed in feces and manure samples for which the host was not determined for a majority of ARG. Among the identified AG-O-PT, the hosts were mostly *Proteobacteria* and a few *Firmicutes* members.

In the betalactam class, among the class A betalactamases, which were detected only in fecal samples, the *blaCFX* ARG were detected only in *Bacteroidetes* members. The other class C betalactamases that were in lower abundance were *blaACI,* detected in *Firmicutes*, and *blaCTX*, detected in *Proteobacteria* members. Class D (*blaOXA*) betalactamases that were detected in manure and lagoon samples were mostly identified in *Bacteroidetes* and *Proteobacteria* members. The bacterial hosts carrying ARG conferring resistance to glycopeptides in soil samples were identified as *Actinobacteria* members.

Among the ARG conferring resistance to MLS, the *lnuC* gene was the most abundant ARG and was carried predominantly by *Clostridia* and *Bacilli* (*Firmicutes*) followed by *Proteobacteria* and *Spirochaete* members. A small portion of ARG conferring resistance to MLS was also contributed by *lnuA* which was carried by *Bacteroidetes* members in the fecal samples of dairy cows from both farm types. The efflux pumps for macrolides and streptogramins were mostly found in *Bacilli* and *Clostridiales* in manure samples. The ARG responsible for efflux pump-based resistance in the multi-drug resistance category were mostly identified in *Proteobacteria*. The bacterial hosts for ARG conferring resistance to rifampin (*rpoB*) were mostly *Actinobacteria* followed by *Firmicutes* in feces from all animal groups, whereas the *rph* ARG identified in soil samples were carried by *Actinobacteria* members.

Among the genes conferring resistance to tetracyclines, the bacterial hosts for *tet32* and *tet40* detected in feces from all animal groups were *Firmicutes* members, *tet36* and *tetQ* were specifically found in *Bacteroidetes* members, *tet44* and *tetT* were found in *Firmicutes* and *Proteobacteria* members, and *tetW* was detected in several unidentified phyla and across lineages of *Firmicutes*, *Proteobacteria*, and *Bacteroidetes*. The *tetR* and *tetY* genes identified in manure samples was restricted to *Proteobacteria* members only. The *sulI* and *sulII* ARG conferring resistance to sulfonamides were also identified in *Proteobacteria* lineages only.

## Discussion

There is strong evidence that livestock and their environments have become large reservoirs for ARB and ARG [18, 21]. As the frequent use of antimicrobials in livestock has been listed as one of the major causal factors, adhering to organic standards by restricting antimicrobial use [33] may prevent or reduce AMR dissemination on farms [34]. The purpose of this study was to determine whether there were differences in the distribution of microbiota and ARG on dairy farms that differed in their use of antimicrobials. Overall, we found that the abundance and diversity of ARG was much higher in the conventional herds compared to organic herds. Feces in particular had a higher concentration of commonly detected ARG and those that conferred resistance to some of the critically and highly important antimicrobials used to treat human infections. Similar patterns were reported by Rovira et al. [35], who found that a higher number of ARG were detected in feces and waste water samples in conventional herds compared to organic herds, although the number of unique ARG identified in this study was lower (95 vs. 144) than those reported in Rovira et al. [35].

At the community level, composition of microbiomes and resistomes did not differ between organic and conventionally managed dairy herds. We found that the microbiome and resistome differed between sample types (*P* < 0.05) which agrees with our previous findings [21] and those of Noyes et al. [13], where both studies investigated different agricultural sites and reported that the associated respective resistomes were different. It was reported by Wang et al. [36] that changes in the core resistome identified in manure samples were highly correlated with the microbial phylogeny of the same sample indicating that presence and activity of ARG may be influenced by microbial populations in the samples. Similar clustering patterns between microbiomes and resistomes of the same sample types as observed in this study may indicate that phylogenetic composition of the microbiome has an influence on ARG profiles.

Within each sample type, fecal resistomes differed by farm type in their relative abundance and distribution, but no such differences were observed for manure and soil samples, suggesting that overall differences in resistomes between organic and conventional farms were driven mostly by fecal resistomes and not those from manure and soil samples. The type of antimicrobials that were administered on the 3 conventional herds in this study were recorded as cephalosporins, penicillins, and streptomycins for dry cow therapy; cephalosporins for mastitis; macrolides, fluoroquinolones, and chloramphenicols for pneumonia; and ampicillin and cephalosporins for metritis (Additional Table 1). Accordingly, feces from conventional animal groups had diverse (198 out of 401 unique ARG) ARG that contributed to a higher abundance (total ARG counts: 6037 vs.1429) compared to their counterparts from organic herds. Among the animal groups, feces from non-lactating cows and heifers had a higher abundance of ARG compared to lactating cows in all 3 conventional dairy herds. Feces from all animals had ARG conferring resistance to cephalosporin (*cfx, ctx*), penicillins (*ctx, rob, aci, cblA*), MLS, and phenicols. These results are in agreement with those of Rovira et al. [35] indicating that ARG abundance in fecal samples correspond to the types of antimicrobials administered to animals. Lactating cows from conventional dairy herds that were sampled for this study had not been given antimicrobials at the time of sampling, and this may explain why the ARG abundance and diversity was lower in feces from lactating cows than those from non-lactating cows and heifers. We also found that feces from the heifers in the medium conventional herd had an exceptionally higher abundance and greater diversity of ARG than other animal groups across all conventional herds. The exact cause for this high abundance of ARG in heifers on this farm is beyond the scope of this study. The fecal samples from individual cows were pooled by type and farm for this study and therefore it is not clear if one or more cows were enriched in ARG. Furthermore, heifers from this particular farm were raised on a different site for efficient use of feed and other resources.

Among the organic herds, the large and medium herds stopped using antimicrobials more than 20 years prior to sample collection for this study and the small herd had not used antimicrobials since 2013. Fecal samples from all animal groups from both conventional and organic herds had ARG conferring resistance to betalactamases and MLS as well as tetracycline ribosomal protection proteins (*tetW, tet32*, and *tet44*), indicating that these may form the core resistome in dairy cow feces. Fecal samples from animal groups from organic herds were relatively clean compared to those from conventional herds, particularly non-lactating cows and heifers. Lactating cows from the large and medium organic herds had ARG conferring resistance to rifampins in fecal samples which were not detected in other animal groups but were detected at much lower concentration in conventional herds. Rifampin is used to treat certain types of infections in horses [37] and is occasionally used in dogs and cats [38] but is not prescribed for use in livestock. It has been reported that ARG conferring resistance to rifampin, particularly *rpoB*, contribute to 35% of the abundance of annotated ARG across diverse environmental samples such as aquaculture sediment, sludge, biofilm, and river water environments [39]. It is interesting to note that the microbiome of lactating cows on organic herds for unknown reasons may select for bacteria with rifampin resistance. Rifampin is a broad-spectrum antimicrobial that is the drug of choice for the treatment of tuberculosis in humans. Therefore, the findings of this study highlight the need for further investigations into the prevalence, persistence, distribution, and functional role of ARG conferring resistance to rifampin on organic farms. Furthermore, while the bacterial hosts that carried these genes were identified as *Clostridia* in feces and *Gammaproteobacteria* members in manure and lagoon samples, several of the bacterial hosts carrying rifampin resistance are yet to be identified reinforcing the need for further research on rifampin resistance. In contrast, lactating cows from the small organic herd did not have ARG conferring resistance to rifampin in their fecal samples but carried low numbers of ARG conferring resistance to aminoglycosides and class A betalactamases. It is not clear if the history of withholding antimicrobials only for 4 years or other management factors on this farm contributed to low levels of ARG conferring resistance to commonly used antimicrobials in feces. Collectively, these data indicate the need for research on the functional role of ARG on organic operations and how these functions change with different bacterial hosts.

The ARG profiles of manure and lagoon samples between conventional and organic herds were similar, although the organic herds had lower abundance than the conventional herds. We have reported that similar to feces, manure is a hotspot for ARG and is enriched in a diverse ARG gene pool [21] which is in agreement with this study. A history of organic management practices on farms did not seem to affect the ARG profiles of manure or lagoon samples in organic herds. There were ARG conferring resistance to multiple antimicrobials including aminoglycosides, betalactamases (particularly class D), MLS, multidrug resistance, cationic antimicrobial peptides, phenicols, sulfonamides, and tetracyclines. The presence of extended-spectrum ARG conferring resistance to betalactamases, such as *blaOXA*, a class D betalactamase, on dairy sectors at a very low abundance in this study was also reported by Rovira et al. [35] and Vikram et al. [40] using metagenomic approaches. Interestingly, we found that most of these betalactamases were identified only in *Bacteroides spp* in this study. The *Bacteroides* group of bacteria carries the most unique and diverse resistance mechanisms [41] and the occurrence of ARG conferring resistance to betalactamases in these bacteria in diverse environments has been increasing. The *blaCFXA* gene has been reported in *Bacteroides* isolates from human infections and also in the *Bacteroides* genus identified in cows treated with ceftiofur [42]. The *blaCFXA* gene was found to be highly resistant to first and second generation cephalosporins and moderately resistant to third and fourth generation cephalosporins [43].

Among the MLS resistance genes, it is interesting to note that in feces, commensal bacteria such as members of *Bacteroidetes* and *Firmicutes* carried ARG conferring resistance to lincosamide antimicrobials whereas in manure samples, ARG conferring resistance to macrolides, lincosamides, and streptogramins were detected. Interestingly, only *Bacilli* in manure samples carried ARG for streptogramins. The ubiquitous presence of ARG conferring resistance to MLS in beef cattle and their environment has been documented [44], although these MLS resistance genes can also occur frequently by mutation [45]. Nevertheless, differences in resistance mechanisms between feces and manure for MLS resistance needs further research and may shed light on the prevalence and dissemination pathways of MLS resistance in livestock farm environments.

Across both farm types, soil samples were enriched in genes conferring resistance to aminocoumarins, glycopeptides, multidrug resistance, rifampin, tetracyclines, and trimethoprim. Among soil samples, the sample from the small organic herd had a diverse pool of ARG including efflux pumps for multidrug resistance, MLS, rifampins, and tetracyclines. As per the history obtained from producers, the soils sampled during spring/summer received manure application about 6–9 months prior to sampling time thus indicating the influence of environmental factors on the distribution of diverse ARG in the soil sample; this is in agreement with the findings of Hurst et al. [46] who reported that the abundance of ARG can be influenced by environmental factors such as location, antimicrobial use, farm type, antimicrobial concentrations, and storage of manure samples.

The ubiquitous distribution of tetracycline resistance in cows and their excrements has been well documented [21, 42]. There is evidence to show that the presence of these ARG even in the absence of tetracycline administration is linked to improved health and growth promotion of dairy cattle [47]. These authors stated that *tetO*, *tetW*, and *tetQ* constitute a core tetracycline-specific resistome in heifers and their excrements. Similar findings were also observed in the current study where *tetW* and *tetQ* were among the most abundant ARG across most fecal, manure, and lagoon samples, suggesting that these genes co-occur with other genes or are needed for microbial metabolism. Notably, the similarity between specific gene sequences in *Butyrivibrio*, a commensal rumen bacterial genus, and the *tetO* gene in *Streptococcus pyogenes* indicate a possible transfer of these ARG between microbes of different origin. Further, *tetW* in *Butyrivibrio* has a higher G + C content than its genome, once again suggesting its possible acquisition from other higher G + C genomes [48]. Interestingly, we found that bacterial hosts carrying *tetQ* were *Bacteroidetes* members and those carrying *tetW* were mostly *Clostridia* members, whereas other ARG conferring resistance to tetracycline were detected in *Enterobacteriaceae* members of *Proteobacteria*. The *tet36* and *tet39* ARG were unique to manure and lagoon samples and were identified only in *Bacteroides spp* and *Acinetobacter baumanii,* respectively. The detection of the *tet39* ARG on plasmids of several strains of *Acinetobacter* may indicate its possible dissemination to other Gram-negative bacteria [49] whereas the *tet36* gene, a ribosomal protection gene, failed to disseminate between different species of *Bacteroides* under laboratory conditions [50]. Collectively, these data reveal that while a majority of ARG conferring resistance to tetracycline have become a part of the genomes of commensal bacteria there may be other ARG, such as *tet36* and *tet39*, that are restricted to only a few species. Further investigations are needed to understand the prevalence, persistence, function, and dissemination mechanisms of these ARG.

## Conclusions

Microbial community composition was influenced by sample type but no significant differences were observed between conventional and organic farms for each sample type. The use of antimicrobials had an independent effect on distribution of ARG. Antimicrobial use on farms influenced the distribution of specific ARG such as those conferring resistance to betalactams, tetracyclines, and MLS in conventionally managed cows. These ARG were detected in organically managed operations but at a very low abundance and were carried in commensal bacteria. Irrespective of farm type, all manure and lagoon samples had diverse ARG revealing that antimicrobial use has no effect on the distribution of resistomes in manure samples. All soil samples appeared to have a core resistome that may be highly conserved; however, the total abundance and distribution of ARG may be influenced by environmental factors. Further studies are needed to investigate the prevalence, distribution, and functional significance of ARG on organic farms both on temporal and spatial scales.

## Supplementary Information

**Additional file 1 Table S1**. Selected antimicrobials used on conventional dairy herds.

**Additional file 2: Table S2**. Samples and sequencing information for organic and conventional farms. Ref: reference; Lact: lactating cow; NonLact: non-lactating cow; Org: organic; Conv: conventional.

**Additional file 3: Table S3**. Results of PERMANOVA analysis for organic and conventional farms. R^2^ and *P* values given for farm type, sample type, and their interaction for microbiome and resistome.

**Additional file 4: Table S4**. Distribution of antimicrobial resistance genes (ARG) on organic and conventional farms at 80% gene content.

**Additional file 5: Table S5**. Distribution of antimicrobial resistance genes (ARG) on organic and conventional farms at 100% gene content.

## Data Availability

The datasets generated and/or analysed during the current study are available in the National Center for Biotechnology Information (NCBI) Sequence Read Archive (SRA) under accession number PRJNA588263.

## Abbreviations

AG-O-NT: Aminoglycoside-O-nucleotidyltransferases
AG-O-PT: Aminoglycoside-O-phosphotransferases
AMR: Antimicrobial resistance
ARB: Antimicrobial resistant bacteria
ARG: Antimicrobial resistance genes
MDR: Multi-drug resistance
MLS: Macrolide-lincosamide-streptogramin
SNP: Single nucleotide polymorphism
