# Supplementary material for: The distribution of microbiomes and resistomes across farm environments in conventional and organic dairy herds in Pennsylvania
Source: Environ Microbiome. 2020 Dec 9;15:21. doi: 10.1186/s40793-020-00368-5 (PMC8066844; doi:10.1186/s40793-020-00368-5)
Supplement: Supplementary file 1 — Additional file 1 Table S1. Selected antimicrobials used on conventional dairy herds. [file 40793_2020_368_MOESM1_ESM.docx]

| **Farm** | **Antimicrobial** | **Class** | **Target Animal and/or Condition** |
| --- | --- | --- | --- |
| Small conventional | Ceftiofur | Cephalosporin | Dry cow therapy  Mastitis |
|  | Enrofloxacin | Fluoroquinolone | Calf pneumonia |
|  | Ampicillin | Penicillin | Metritis |
| Medium conventional | Cephapirin benzathine | Cephalosporin | Dry cows |
|  | Ceftiofur | Cephalosporin | Mastitis  Metritis |
|  | Ampicillin | Penicillin | Metritis |
|  | Florfenicol | Amphenicol | Calf pneumonia |
| Large conventional | Penicillin | Penicillin | Dry cow therapy |
|  | Ceftiofur | Cephalosporin | Mastitis  Metritis |
|  | Ampicillin | Penicillin | Metritis |
|  | Tulathromycin | Macrolide | Calf pneumonia |

**Additional Table 1. Selected antimicrobials used on conventional dairy herds.**
