# Supplementary material for: The distribution of microbiomes and resistomes across farm environments in conventional and organic dairy herds in Pennsylvania
Source: Environ Microbiome. 2020 Dec 9;15:21. doi: 10.1186/s40793-020-00368-5 (PMC8066844; doi:10.1186/s40793-020-00368-5)
Supplement: Supplementary file 3 — Additional file 3: Table S3. Results of PERMANOVA analysis for organic and conventional farms. R2 and P values given for farm type, sample type, and their interaction for microbiome and resistome. [file 40793_2020_368_MOESM3_ESM.docx]

|  |  | Microbiome | | Resistome | |
| --- | --- | --- | --- | --- | --- |
|  |  | R^2^ | *P* value | R^2^ | *P* value |
| PERMANOVA | Farm Type | 0.05546 | 0.357 | 0.04289 | 0.732 |
|  | Sample Type | 0.24644 | 0.004 | 0.17578 | 0.044 |
|  | Farm Type: Sample Type | 0.08069 | 0.417 | 0.03703 | 0.962 |
